# Evaluation of *Stratiolaelaps scimitus* (Acari: Laelapidae) for controlling the root-knot nematode, *Meloidogyne incognita* (Tylenchida: Heteroderidae)

**DOI:** 10.1038/s41598-020-62643-2

**Published:** 2020-03-27

**Authors:** Si-Hua Yang, Dan Wang, Chun Chen, Chun-Ling Xu, Hui Xie

**Affiliations:** Laboratory of Plant Nematology and Research Center of Nematodes of Plant Quarantine, Department of Plant Pathology/Guangdong Province Key Laboratory of Microbial Signals and Disease Control, College of Agriculture, South China Agricultural University, Guangzhou, People’s Republic of China

**Keywords:** Microbiology techniques, Agroecology, Microbial ecology, Ecology, Agroecology, Behavioural ecology

## Abstract

Root-knot nematodes are one of the most harmful plant-parasitic nematodes (PPNs). In this paper, the predation of *Stratiolaelaps scimitus* against *Meloidogyne incognita* was tested in an individual arena, and the control efficiency of the mite on the nematode in the water spinach (*Ipomoea aquatica*) rhizosphere was studied with a pot experiment. The results showed that *S. scimitus* could develop normally and complete its life cycle by feeding on second-stage juveniles of *M. incognita* (*Mi*-J2). The consumption rate of a 24 h starving female mite on *Mi*-J2 increased with the increase of prey density at 25 °C. Among the starvation treatments, the nematode consumption rate of a female mite starved for 96 h at 25 °C was highest; and among temperature treatments, the maximum consumption rate of a 24 h starving female mite on *Mi*-J2 was at 28 °C. The number of *M. incognita* in the spinach rhizosphere could be reduced effectively by releasing *S. scimitus* into rhizosphere soil, and 400 mites per pot was the optimum releasing density in which the numbers of root knots and egg masses decreased by 50.9% and 62.8%, respectively. Though we have gained a greater understanding of *S. scimitus* as a predator of *M. incognita*, the biocontrol of *M. incognita* using *S. scimitus* under field conditions remains unknown and requires further study.

## Introduction

Root-knot nematodes, *Meloidogyne* spp., are an economically important obligate parasites of plant root^1^ and parasitize more than 3000 species of plant^2^. The losses that are caused by root-knot nematode to crops can be up to 87–100% when root-knot nematodes are in co-presence with other pathogens^3^, with four major species: *M. arenaria*, *M. hapla*, *M. incognita*, and *M. javanica*^3,4^. In tropical and subtropical areas with abundant rainfall and a mild climate, root-knot nematodes are particularly harmful. After the occurrence of root-knot nematode diseases, the yield is generally reduced by 10–20%, and the severity is more than 75%^5^. In China, root-knot nematodes can damage about 50% of greenhouse vegetables and cause annual economic losses of approximately 400 million dollars^6^. Currently, root-knot nematodes are becoming increasingly destructive pests on greenhouse vegetables worldwide^4,7,8^.

Chemical control has been the fastest and most effective way to control plant nematodes for decades^4^. However, chemical nematicides are high toxic and high residual, and their long-term use would cause serious environmental pollution and other adverse effects^9^. Thus, reducing the use of chemical pesticides has become a trend, and it is urgent to develop safe, effective and sustainable biological control methods. Biological control of plant-parasitic nematodes (PPNs) refers to the use of natural enemies to prey PPNs or otherwise weaken their infestation or viability, thereby achieving the purpose of plant protection^10^. Among the natural enemies of plant nematodes, the most studied were nematophagous fungi^11^, followed by *Pasteuria penetrans*^12^ and rhizobacteria^13,14^. Later studies have shown that some predatory mites can prey on plant-parasitic nematodes (PPNs), such as *Pergalumna* sp. on *Pratylenchus coffeae* and the second-stage juveniles of *M. javanica*^15^; *Sancassania* (Caloglyphus) *berlesei* on the egg masses of *Meloidogyne* spp.^16^; *Tyrophagus putrescentiae* on the egg masses or adult females of *M. incognita*^17^; *Neoseiulus barkeri* on the second-stage juveniles of *M. incognita* (*Mi*-J2)^18,19^ and adult females of *Radopholus similis*^19^; *Blattisocius dolichus* on *Mi*-J2^8^, and so on. *Stratiolaelaps scimitus* is a generalist predatory mite living in the soil and rhizosphere of plants^20–22^ and can prey on harmful insects, such as fungus gnats and thrips^23,24^. However, there is no report of *S. scimitus* preying on nematodes. In this paper, the development and reproduction of *S. scimitus* feeding on *Mi*-J2 were compared to those feeding on *T. putrescentiae*, a prey mite commonly used for experimental and commercial rearing of *S. scimitus*^20,21,25–27^. Moreover, the predation and controlling effects of *S. scimitus* on *M. incognita* were studied in the arena and with pot experiments, respectively, to determine whether *S. scimitus* could be used as a predatory natural enemy of root-knot nematodes and whether *S. scimitus* has value and the potential for application in the control of root-knot nematode disease. The research results provide a scientific basis for further use of predatory mites to control nematodes.

## Results

### Reproductive life table of *S. scimitus*

**Figure 1:**
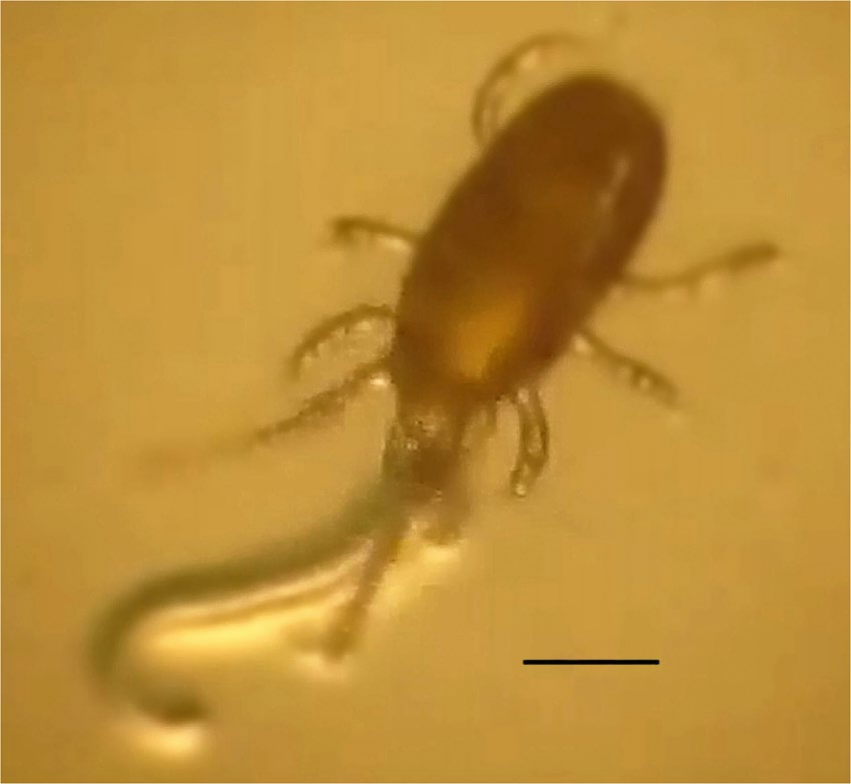
A female *Stratiolaelaps scimitus* predating on second-stage juveniles of *Meloidogyne incognita*. Scale bars = 0.02 mm.

The results of the experiment showed that the experimental population of *S. scimitus* could normally develop and complete its life cycle, including five developmental stages: eggs, larvae, protonymph, deutonymph and adult, when fed with *Mi*-J2 (Fig. 1, Table 1). In addition, female adult mites could survive for 17–24 days after they stopped laying eggs, and then experience parthenogenesis. There was no significant difference between *Mi*-J2 and *T. putrescentiae* with respect to the duration of development of the mite (t_eggs_ = 0.424, t_larvae_ = −0.789, t_protonymph_ = 0.675, t_deutonymph_ = −0.16, t_adult_ = 0.698, df = 38, p > 0.05). Compared with the treatment of preying on *T. putrescentiae*, the pre-oviposition period of *S. scimitus* fed on *Mi*-J2 was significantly longer (t = −5.325, df = 38, p < 0.05), the daily and total egg production of each female mite were larger, but with no significant difference (t_daily eggs_ = −1.993, t_total eggs_ = −0.912, df = 38, p > 0.05), and the lifespan, oviposition period and post-oviposition period of female mites had no significant difference either (t_lifespan_ = 0.798, t_oviposition_ = 1.358, t_post-oviposition_ = 0.330, df = 38, p > 0.05) (Table 2).

**Table 1:** The developmental duration of *Stratiolaelaps scimitus* fed on second-stage juveniles of *Meloidogyne incognita* and *Tyrophagus putrescentiae*.

Prey	Developmental duration (d)				
	Eggs	Larvae	Protonymph	Deutonymph	Adult
*Mi*-J2	3.18 ± 0.12a	1.18 ± 0.07a	3.48 ± 0.13a	3.40 ± 0.09a	72.15 ± 0.69a
*T. putrescentiae*	3.25 ± 0.13a	1.10 ± 0.07a	3.60 ± 0.13a	3.38 ± 0.13a	72.8 ± 0.64a

The data are presented as the mean ± standard error of five replicates; the same lowercase letters in every column indicate that the means are not significantly different at the 0.05 level (t test).

**Table 2:** The spawning duration, lifespan and spawning amount of a female *Stratiolaelaps scimitus* fed on second-stage juveniles of *Meloidogyne incognita* and *Tyrophagus putrescentiae*.

Parameter	*Mi*-J2	*T. putrescentiae*
Pre-oviposition	3.80 ± 0.15b	2.80 ± 0.12a
Oviposition	47.75 ± 0.75a	49.20 ± 0.76a
Post-oviposition	20.60 ± 0.41a	20.80 ± 0.44a
Lifespan	83.38 ± 0.68a	84.13 ± 0.65a
Total eggs	92.80 ± 1.23a	91.05 ± 1.47a
Daily eggs	1.95 ± 0.03a	1.86 ± 0.03a

The data are presented as the mean ± standard error of five replicates; the same lowercase letters in every row indicate that the means are not significantly different at the 0.05 level (t test).

According to the survival rate of female adult mites and the average number of female offspring per female adult mite that were obtained by the experiment, the reproductive life tables of *S. scimitus* fed on *Mi*-J2 and *T. putrescentiae* were calculated (Tables 1 and 2 in Additional File). Then, the life table parameters of *S. scimitus* were calculated from the reproductive life table (Table 3). The intrinsic rate of increase (r_m_) and the finite rate of increase (λ) of *S. scimitus* fed on *Mi*-J2 were 0.6335 day^−1^ and 1.8843, respectively; both of them were greater than those of *S. scimitus* fed on *T. putrescentiae* (0.5026 day^−1^ and 1.6530). The population doubling time (Dt) of *S. scimitus* fed on *Mi*-J2 (1.0941 days) was less than that of *S. scimitus* fed on *T. putrescentiae* (1.3791 days).

**Table 3:** The life table parameters of *Stratiolaelaps scimitus* fed on second-stage juveniles of *Meloidogyne incognita* and *Tyrophagus putrescentiae*.

Parameter	Prey	
	*Mi*-J2	*T. putrescentiae*
The net reproductive rate - R_0_	66.4381	57.1218
The mean generation time - T (days)	35.2595	34.5559
The intrinsic rate of increase - r_m_ (day^−1^)	0.6335	0.5026
The finite rate of increase - λ	1.8843	1.6530
The population doubling time - Dt (days)	1.0941	1.3791

### Predation of *S. scimitus* on *Mi*-J2 at various prey densities, temperatures and starvation times

**Figure 2:**
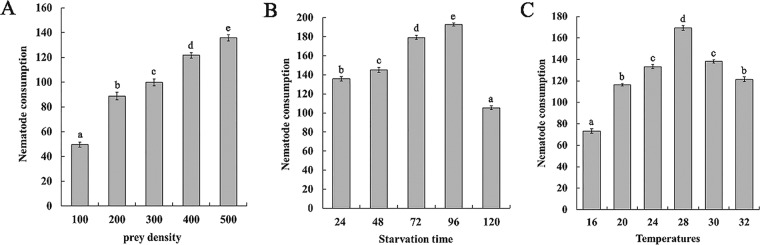
Daily consumption of a female *Stratiolaelaps scimitus* on second-stage juveniles of *Meloidogyne incognita*. (**A**) Daily consumption rates of *S. scimitus* on *Mi*-J2 at various prey densities. (**B**) Daily consumption of *S. scimitus* on *Mi*-J2 at various starvation times. (**C**) Daily consumption of *S. scimitus* on *Mi*-J2 at various temperatures. The same lowercase letters in each figure indicate that the means are not significantly different (P > 0.05) to those obtained by Tukey’s test.

The number of nematodes consumed by *S. scimitus* with 24 h starvation under different prey densities showed that with an increase of the prey density, consumption significantly increased (F = 166.34 df = 4,20, p < 0.05); the maximum consumption was 135.8 nematodes at 500 nematodes per arena. However, with an increase of the prey density, consumption rates gradually plateaued from 100 to 500 (Fig. 2A).

With 500 *Mi*-J2 per arena, the consumption rate of *S. scimitus* on *Mi-*J2 was affected by the starvation time and temperature (Fig. 2B,C). With the starvation treatment time ranging from 24 to 96 h, consumption increased significantly with the prolongation of starvation (F = 151.99 df = 3,16, p < 0.05). However, when the starvation time was prolonged to 120 h, consumption decreased significantly (F = 10.46.41 df = 1,8, p < 0.05). Therefore, the maximum consumption of *S. scimitus* on *Mi*-J2 was 193 nematodes when starved for 96 h. In the temperature range of 16 to 28 °C, the consumption of *S. scimitus* on *Mi*-J2 increased significantly with the increasing temperature (F = 415.85 df = 3,16, p < 0.05). However, in the temperature range of 28 to 32 °C, consumption decreased significantly as the temperature increased (F = 142.95 df = 2,12, p < 0.05). Therefore, the optimum predatory temperature for *S. scimitus* preying on *Mi*-J2 was 28 °C, and consumption at this temperature was 169 nematodes.

### The controlling effect of *S. scimitus* on *M. incognita* in the rhizosphere of spinach

**Figure 3:**
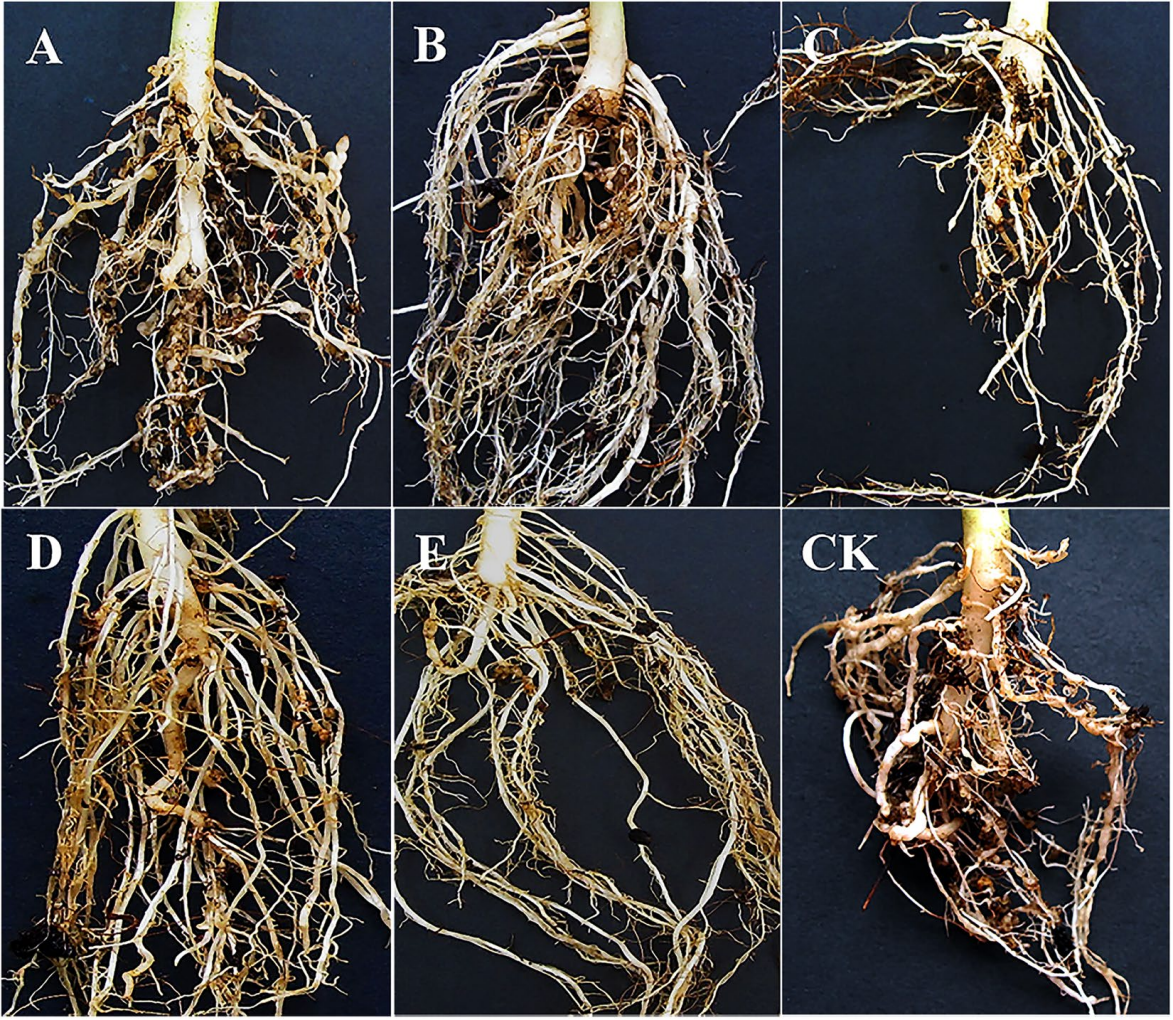
Growth of spinach roots after different treatments. **(A–E**) Treatment of 100, 200, 300, 400 and 500 *Stratiolaelaps scimitus* released into the rhizosphere soil per pot for 30 days after inoculation with *Mi*-J2 for ten days. (**CK**) Control treatment with inoculated nematodes alone.

The pot experiment results of releasing *S. scimitus* to control *M. incognita* in the rhizosphere of spinach showed that mites could reduce the number of root-knot nematodes and mitigate the harm they cause to the host (Fig. 3). The number of root-knots in spinach roots with mites was significantly less than that of CK (Table 4). Within mites that were released in the range of 100 to 500, the number of root-knots decreased with the increase of mites. There was no significant difference between releasing 400 and 500 mites (F = 1.88 df = 1,8, p > 0.05), but there was a significant difference among other mite-releasing treatments (F = 149.58 df = 5,24, p < 0.05). The number of egg masses of *M. incognita* in spinach roots with mites was significantly less than that of CK (F = 23.63 df = 5,24, p < 0.05) except when 100 mites were released (F = 1.81 df = 1,8, p > 0.05). The number of egg masses decreased as the number of mites that were released increased. The height of the spinach with mites was significantly higher than that of CK (F = 61.19 df = 5,24, p < 0.05), except when 100 mites were released (F = 0.75, df = 1,8, p > 0.05), which was proportional to the number of released mites.

**Table 4:** The number of root-knot and egg masses in roots and the plant heights of the potted spinach at 30 days after the release of *Stratiolaelaps scimitus* into rhizosphere soil inoculated with 2000 *Mi*-J2 for ten days.

Treatments	Plant height (cm)	Root-knot number	Egg masses number
100*	35.85 + 1.01a	280.60 + 5.67d	60.40 + 4.71 cd
200*	45.77 + 1.81b	247.40 + 5.48c	47.40 + 5.52bc
300*	51.27 + 1.71b	216.20 + 6.85b	36.80 + 3.17ab
400*	61.32 + 1.27c	163.20 + 5.64a	25.60 + 2.27a
500*	66.80 + 1.97c	153.00 + 4.85a	22.80 + 1.66a
CK	33.78 + 2.16a	332.20 + 5.19e	68.80 + 4.10d

The data are presented as the mean ± standard error of five replicates; the same lowercase letters in every column indicate that the means are not significantly different at the 0.05 level (Tukey’s test). *Number of mites released into the rhizosphere. CK: seedling inoculated with nematodes alone.

## Discussion

When *S. scimitus* fed on *Mi*-J2 and *T. putrescentiae*, they could develop normally and complete their life cycles, and there was no significant difference in their lifespans. However, *S. scimitus* had higher fertility, r_m_, and λ, and shorter Dt when feeding on *Mi*-J2 than when feeding on *T. putrescentiae*, which suggests that *Mi*-J2 are more suitable for the development and reproduction of the mite. Compared with the reports of Cabrera *et al*.^22^, when feeding on potworms (*r*_m_ = 0.142 day^−1^, λ = 1.153, Dt = 4.85 days) and fungus gnat (*Bradysia* aff*. coprophila*) larvae (*r*_m_ = 0.105 day^−1^, λ = 1.110, Dt = 6.58 days), *S. scimitus* had higher *r*_m_ (0.6335 day^−1^) and λ (1.8843), and shorter Dt (1.0941 days) when feeding on *Mi*-J2. Therefore, when *Mi*-J2 were used as prey, *S. scimitus* had a strong incremental ability and a rapid reproductive rate.

The net reproductive rate (R_0_) means that the number of individuals in a population increases to an average multiple after a generation. In this study, the R_0_ of *S. scimitus* that were fed on *Mi*-J2 was 66.4 after one generation (R_0_ = 66.4), which was not only higher than that of mites fed on *T. putrescentiae* (R_0_ = 57.1) but was also higher than that of mites fed on potworms (R_0_ = 14.5) and fungus gnats (R_0_ = 17.3)^22^. Abou El-Atta *et al*.^16^ reported that the intrinsic rate of the increase of *S. berlesei* fed on the eggs of *Meloidogyne* spp. was higher at 25 and 30 °C (r_m_ was 0.23 day^−1^ and 0.29 day^−1^, respectively), which could be a result of the short generation times at those temperatures. However, the intrinsic rate of the increase of *S. scimitus* fed on *M. incognita* was 0.6335 day^−1^ at 25 °C, which was 2.75 times higher than that of *S. berlesei* fed on root-knot nematodes at 25 °C (r_m_ was 0.23 day^−1^)^16^. Therefore, *S. scimitus* has better application potential in the biological control of root-knot nematodes.

Predator-prey interaction is a process of interaction between predators and prey in the environment that is affected by the density of prey, the starvation time of predators and the temperature of the environment. The results of this study showed that the predation of *S. scimitus* on *Mi*-J2 increased with the increase of nematode density within a certain range, which was similar to that of *H. calcuttaensis*^28^ and *Blattisocius dolichus*^8^. This may be due to the increased probability of predator-prey contact. In this study, the number of nematodes consumed by *S. scimitus* was largest after 96 hours of starvation, and the number of nematodes consumed by the mite decreased significantly after more than 96 hours of starvation. Xu *et al*.^8^ reported that *B. dolichus* also consumed the largest amount of *Mi*-J2 at 96 hours of starvation and that the number of nematodes consumed by *B. dolichus* decreased significantly after 96 hours of starvation. This may be due to hunger over a certain period of time; as predatory mites consumed too much energy, their ability to move, search, and dispose of prey also decreased. In this study, the number of *Mi*-J2 consumed by *S. scimitus* was the largest at 28 °C, so this temperature was the best preying temperature for *S. scimitus*. It has been reported that the optimum temperature ranges for the growth and development of *S. scimitus* and *M. incognita* are 24 to 28 °C^29^ and 25 to 30 °C^30^, respectively, which is basically consistent with the optimum temperatures for *S. scimitus* to prey on *Mi*-J2. This will be beneficial to the application of *S. scimitus* in the biological control of *M. incognita* and its improved performance in the field.

In pot experiments, a certain amount of *S. scimitus* was released into the rhizosphere soil of plants where root-knot nematodes occurred, which could effectively reduce the number of root-knot nematodes and significantly reduce the degree of root damage. When 100 mites were released into the rhizosphere soil of the spinach at the initial stage of root-knot nematode infection, the number of root-knots decreased significantly, but there were no significant differences in the number of egg masses or in the height of plant compared with those of non-releasing mites. When the releasing number of mites was 200 in the rhizosphere soil, the number of egg masses began to decrease significantly and the plant height began to increase significantly. This result is consistent with the results of controlling the rhizosphere root-knot nematodes of spinach with the release of *B. dolichus*, as reported by the study of Xu *et al*.^8^ on controlling the rhizosphere root-knot nematodes of spinach with the release of *B. dolichus*. This may be because a certain number of mites can effectively control nematodes. Xu *et al*.^8^ used *B. dolichus* to control the root-knot nematodes of spinach in pots. In their experiment, the optimum release number of *B. dolichus* was 500 per pot, which reduced the number of root-knots by 37.1% and the number of egg masses by 55.1%, and when 400 mites per pot were released, the number of root-knots and egg masses decreased by 32.5% and 46.8%, respectively. In this experiment, there was no significant difference between the effect of releasing 400 and 500 mites per pot. Thus, it was considered that releasing 400 mites per pot was the best releasing density by which to control root-knot nematodes, which reduced the number of root knots and egg masses by 50.9% and 62.8%, respectively. These results indicate that *S. scimitus* have a better control effect on root-knot nematodes, and may be a potentially effective natural enemy for the biocontrol of *M. incognita*. Moreover, in the pot experiment we sterilized the soil to eliminate the effect of other organisms (e.g. other mites and free-living nematodes).

## Conclusion

In conclusion, *S. scimitus* could develop normally and complete its life cycle by feeding on *M. incognita*. It has a strong predatory ability on controlling *M. incognita* and is an effective natural enemy of nematodes. Moreover, this is a preliminary study that was conducted in a laboratory to determinate the predation of mites on nematodes under stable soil conditions. Thus, although we have gained a greater understanding of *S. scimitus* as a predator of *M. incognita*, the biocontrol of *M. incognita* using this mite under field conditions remains unknown and requires further study.

## Materials and methods

### Biological materials

The *M. incognita* used in this study was isolated as described by Barker^31^ and identified by morphological analysis as described by Hunt and Handoo^32^ in Plant Nematology Laboratory, South China Agricultural University (SCAU), Guangzhou, China. It was preserved on the roots of Thai white bone willow leaf water spinach (*Ipomoea aquatica*) in the greenhouse. Egg masses were selected from the roots of spinach inoculated with *M. incognita* and formed root-knots. They were incubated in a dish containing sterile water, and *Mi*-J2 were obtained one week later. Both *S. scimitus* and *T. putrescentiae* were obtained from the Institute of Plant Protection, Chinese Academy of Agricultural Sciences, Beijing, China. The mites were reared in an artificial climate chamber at a temperature of 25 ± 1 °C, with a relative humidity (R.H.) of 80%, and in darkness. The *S. scimitus* were fed with *T. putrescentiae*, while *T. putrescentiae* were fed with wheat bran. The spinach seeds were produced by Thai Kingdom Cai Rongcheng Seed Co., Ltd. The soil medium that was used for the plants was sterilized at 121 °C for 2 h, and the pots (10 cm diameter, 8 cm high) were also sterilized before the water spinach seeds were planted. In addition to pot experiments, other experiments were conducted in an arena, a Petri dishes (35 mm diameter, 15 mm height) filled with 5% sterilized water agar^33^. The arena is a platform for observing and studying the development, reproduction and life table of *S. scimitus* and the predatory effects of mites on nematodes under different conditions,

### Development, reproduction and life table of *S. scimitus*

One hundred female adults *S. scimitus* were selected for egg laying in the arena. The eggs (about 50 eggs), which were laid by the female mites within 12 hours, were collected and fed separately in the arena. Then, they were placed in a dark incubator. The culture conditions were 25 ± 1 °C and 80% R.H. The developmental duration and survival were observed under a stereoscopic microscope every 12 hours. Because the egg and the juvenile mite did not feed, when the egg developed into a nymph, it was moved into a new arena and provided with sufficient amounts of *Mi*-J2 and *T. putrescentiae* as prey in the arena, respectively. The new molting shell could be observed to confirm the onset of a new developmental stage. An identical arena for *S. scimitus* was replaced every 12 h to ensure freshness of the prey. When the eggs of *S. scimitus* developed into adults, the male and female mites were paired, and a male mite was added immediately after the death or loss of the male mite was found to have occurred during the process. The pre-oviposition, oviposition, post-oviposition, daily eggs, and lifespan of the female mites were recorded. Based on the data obtained above, the life table and its parameters of *S. scimitus* fed on *Mi*-J2 and *T. putrescentiae* were calculated as described by Maia *et al*.^34^. Each treatment was repeated 20 times.

### Predation of *S. scimitus* on *Mi*-J2 at various prey densities, temperatures, and starvation times

Through three experiments, the effects of different predation densities, starvation time, and temperature on *S. scimitus* preying on Mi-J2 were studied. (1) Five densities of *Mi*-J2 (100, 200, 300, 400 and 500 per arena) were established; then, an adult female *S. scimitus* with 24 h starvation treatment was introduced into each arena, and the arenas were sealed and cultured in simulated darkness at 25 ± 1 °C. (2) An adult female *S. scimitus*, which was starved for 24, 48, 72, 96 and 120 h, was introduced into each arena with 500 *Mi*-J2; then, the arenas were sealed and cultured in simulated darkness at 25 ± 1 °C. (3) An adult female *S. scimitus* with 24 h starvation treatment was introduced into the arenas with 500 *Mi*-J2; then, the arenas were sealed and cultured in simulated darkness at five temperatures conditions (16, 20, 24, 28, 30 and 32 °C). After 24 hours of culturing, the consumption of nematodes was counted under a stereomicroscope (Model SMZ 745, Nikon)^8^. Each treatment was repeated 5 times.

### Pot experiments

The spinach seeds were seeded in sterile medium soil for 15 d as they developed into seedlings, and the seedlings were transferred to pots containing 0.8 L sterile medium soil^35^. After transplanting for 5 d, 2000 *Mi*-J2 were inoculated in the rhizosphere soil per pot and incubated in a greenhouse at 20–30 °C. Ten days after the inoculation of nematodes, 100, 200, 300, 400 and 500 *S. scimitus* were released into the rhizosphere soil. A disc (diameter 15 cm, depth 3 cm) was placed at the bottom of the pot and water was added to prevent the mites from escaping. For the treatment of inoculating nematodes, no release of mites or other control measures were used as the control (CK). Five replicates were used in each treatment. After releasing mites for 30 days, the spinach seedlings and roots were carefully excavated from the soil, and all the soil from the root were washed under running water. The numbers of root knots, egg masses and plant height were counted.

### Data analysis

The analysis, processing and charting of experimental data were conducted with SPSS19.0 (SPSS Inc., Chicago, IL, USA) and Sigmaplot11.0 (Systat Software Inc., San Jose, CA). The means and standard errors data in this study were compared by means of an analysis of variance (ANOVA); a multiple comparison was tested with Tukey’s test at the 5% level; and a Two-sample comparison was tested with a t test at the 5% level.

### Ethics statement

No specific permissions were required for the nematodes used in this study, and these nematodes were plant pests and not protected by the government.

## Supplementary information

supplementary information.
