# Author Correction: Evaluation of *Stratiolaelaps scimitus* (Acari: Laelapidae) for controlling the root-knot nematode, *Meloidogyne incognita* (Tylenchida: Heteroderidae)

**DOI:** 10.1038/s41598-020-65968-0

**Published:** 2020-06-04

**Authors:** Si-Hua Yang, Dan Wang, Chun Chen, Chun-Ling Xu, Hui Xie

**Affiliations:** Laboratory of Plant Nematology and Research Center of Nematodes of Plant Quarantine, Department of Plant Pathology/Guangdong Province Key Laboratory of Microbial Signals and Disease Control, College of Agriculture, South China Agricultural University, Guangzhou, People’s Republic of China

Correction to: *Scientific Reports* 10.1038/s41598-020-62643-2, published online 27 March 2020

This Article contains errors.

As a result of calculation errors, the intrinsic rate of increase was incorrectly reported in this Article. This, in turn, resulted in errors in the finite rate of increase and population doubling time.

In the Results, under the subheading ‘Reproductive life table of *S. scimitus*’,

“The intrinsic rate of increase (r_m_) and the finite rate of increase (λ) of *S. scimitus* fed on *Mi*-J2 were 0.6335 day^−1^ and 1.8843, respectively; both of them were greater than those of *S. scimitus* fed on *T. putrescentiae* (0.5026 day^−1^ and 1.6530). The population doubling time (Dt) of *S. scimitus* fed on *Mi*-J2 (1.0941 days) was less than that of *S. scimitus* fed on *T. putrescentiae* (1.3791 days).”

should read:

“The intrinsic rate of increase (r_m_) and the finite rate of increase (λ) of *S. scimitus* fed on *Mi*-J2 were 0.1692 day^-1^ and 1.1844, respectively; both of them were greater than those of *S. scimitus* fed on *T. putrescentiae* (0.1619 day^-1^and 1.1757). The population doubling time (Dt) of *S. scimitus* fed on *Mi*-J2 (4.0956 days) was less than that of *S. scimitus* fed on *T. putrescentiae* (4.2822 days).”

In Table 3, the reported values for intrinsic rate of increase, finite rate of increase and population doubling time are incorrect. The corrected Table 3 appears below.

**Table 3:** The life table parameters of *Stratiolaelaps scimitus* fed on second-stage juveniles of *Meloidogyne incognita* and *Tyrophagus putrescentiae*.

Parameter	Prey	
	*Mi*-J2	*T. putrescentiae*
The net reproductive rate - R_0_	66.4381	57.1218
The mean generation time - T (days)	35.2595	34.5559
The intrinsic rate of increase - r_m_ (day^−1^)	0.1692	0.1619
The finite rate of increase - λ	1.1844	1.757
The population doubling time - Dt (days)	4.0956	4.2822

Furthermore, in the Discussion,

“*S. scimitus* had higher *r*_m_ (0.6335 day^−1^) and λ (1.8843), and shorter Dt (1.0941 days) when feeding on *Mi*-J2”

should read:

“*S. scimitus* had higher *r*_m_ (0.1692 day^-1^), and λ (1.1844) and shorter Dt (4.0956 days) when feeding on *Mi*-J2”

Finally, as a result of these changes the following section of the Discussion is no longer correct:

“Abou El-Atta *et al*.^16^ reported that the intrinsic rate of the increase of *S. berlesei* fed on the eggs of *Meloidogyne* spp. was higher at 25 and 30 °C (r_m_ was 0.23 day^−1^ and 0.29 day^−1^, respectively), which could be a result of the short generation times at those temperatures. However, the intrinsic rate of the increase of *S. scimitus* fed on *M. incognita* was 0.6335 day^−1^ at 25 °C, which was 2.75 times higher than that of *S. berlesei* fed on root-knot nematodes at 25 °C (r_m_ was 0.23 day^−1^)^16^.”

Therefore, the second paragraph of the Discussion should read:

“The net reproductive rate (R_0_) means that the number of individuals in a population increases to an average multiple after a generation. In this study, the R_0_ of *S. scimitus* that were fed on *Mi*-J2 was 66.4 after one generation (R_0_  =  66.4), which was not only higher than that of mites fed on *T. putrescentiae* (R_0_  =  57.1) but was also higher than that of mites fed on potworms (R_0_  =  14.5) and fungus gnats (R_0_  =  17.3)^22^. Therefore, *S. scimitus* has better application potential in the biological control of root-knot nematodes.”

