# Supplementary material for: Evaluation of Stratiolaelaps scimitus (Acari: Laelapidae) for controlling the root-knot nematode, Meloidogyne incognita (Tylenchida: Heteroderidae)
Source: Sci Rep. 2020 Mar 27;10:5645. doi: 10.1038/s41598-020-62643-2 (PMC7101305; doi:10.1038/s41598-020-62643-2)
Supplement: Supplementary file 1 — supplementary information. [file 41598_2020_62643_MOESM1_ESM.docx]

**Additional File**

**Table 1** The life table of *Stratiolaelaps scimitus* feeding on the second instar larvae of *Meloidogyne incognita* (25±1℃, 80%R.H., Dark)

| Lifetime x (d) | Survival rate l_x_ | Average production of female m_x_ | l_x_m_x_ | xl_x_m_x_ |
| --- | --- | --- | --- | --- |
| 1.0000 | 1.0000 | immature | immature | immature |
| 2.0000 | 1.0000 | immature | immature | immature |
| 3.0000 | 1.0000 | immature | immature | immature |
| 4.0000 | 0.9545 | immature | immature | immature |
| 5.0000 | 0.9545 | immature | immature | immature |
| 6.0000 | 0.9545 | immature | immature | immature |
| 7.0000 | 0.9545 | immature | immature | immature |
| 8.0000 | 0.9545 | immature | immature | immature |
| 9.0000 | 0.9091 | immature | immature | immature |
| 10.0000 | 0.9091 | immature | immature | immature |
| 11.0000 | 0.9091 | 0.0000 | 0.0000 | 0.0000 |
| 12.0000 | 0.9091 | 0.0000 | 0.0000 | 0.0000 |
| 13.0000 | 0.9091 | 0.0000 | 0.0000 | 0.0000 |
| 14.0000 | 0.9091 | 1.3333 | 1.2121 | 16.9697 |
| 15.0000 | 0.9091 | 1.6250 | 1.4773 | 22.1591 |
| 16.0000 | 0.9091 | 1.8750 | 1.7045 | 27.2727 |
| 17.0000 | 0.9091 | 2.0556 | 1.8687 | 31.7677 |
| 18.0000 | 0.9091 | 2.1000 | 1.9091 | 34.3636 |
| 19.0000 | 0.9091 | 2.1500 | 1.9545 | 37.1364 |
| 20.0000 | 0.9091 | 2.2500 | 2.0455 | 40.9091 |
| 21.0000 | 0.9091 | 1.9500 | 1.7727 | 37.2273 |
| 22.0000 | 0.9091 | 1.9000 | 1.7272 | 38.0000 |
| 23.0000 | 0.9091 | 2.0500 | 1.8636 | 42.8636 |
| 24.0000 | 0.9091 | 1.5500 | 1.4091 | 33.8182 |
| 25.0000 | 0.9091 | 2.1000 | 1.9091 | 47.7273 |
| 26.0000 | 0.9091 | 2.2000 | 2.0000 | 52.0000 |
| 27.0000 | 0.9091 | 1.8500 | 1.6818 | 45.4091 |
| 28.0000 | 0.9091 | 1.9500 | 1.7727 | 49.6364 |
| 29.0000 | 0.9091 | 1.9500 | 1.7727 | 51.4091 |
| 30.0000 | 0.9091 | 2.0500 | 1.8636 | 55.9091 |
| 31.0000 | 0.9091 | 1.8500 | 1.6818 | 52.1364 |
| 32.0000 | 0.9091 | 1.7500 | 1.5909 | 50.9091 |
| 33.0000 | 0.9091 | 1.6000 | 1.4545 | 48.0000 |
| 34.0000 | 0.9091 | 1.7500 | 1.5909 | 54.0909 |
| 35.0000 | 0.9091 | 1.6500 | 1.5000 | 52.5000 |
| 36.0000 | 0.9091 | 1.5500 | 1.4091 | 50.7273 |
| 37.0000 | 0.9091 | 1.5000 | 1.3636 | 50.4545 |
| 38.0000 | 0.9091 | 1.7000 | 1.5455 | 58.7273 |
| 39.0000 | 0.9091 | 1.3500 | 1.2273 | 47.8636 |
| 40.0000 | 0.9091 | 1.4500 | 1.3182 | 52.7273 |
| 41.0000 | 0.9091 | 1.4000 | 1.2727 | 52.1818 |
| 42.0000 | 0.9091 | 1.5000 | 1.3636 | 57.2727 |
| 43.0000 | 0.9091 | 1.2500 | 1.1364 | 48.8636 |
| 44.0000 | 0.9091 | 1.1500 | 1.0455 | 46.0000 |
| 45.0000 | 0.9091 | 1.0500 | 0.9545 | 42.9545 |
| 46.0000 | 0.9091 | 1.1500 | 1.0455 | 48.0909 |
| 47.0000 | 0.9091 | 1.0000 | 0.9091 | 42.7273 |
| 48.0000 | 0.9091 | 0.9500 | 0.8636 | 41.4545 |
| 49.0000 | 0.9091 | 0.9000 | 0.8182 | 40.0909 |
| 50.0000 | 0.9091 | 0.8500 | 0.7727 | 38.6364 |
| 51.0000 | 0.9091 | 0.9000 | 0.8182 | 41.7273 |
| 54.0000 | 0.9091 | 0.7500 | 0.6818 | 36.8182 |
| 55.0000 | 0.9091 | 0.7500 | 0.6818 | 37.5000 |
| 56.0000 | 0.9091 | 0.4000 | 0.3636 | 20.3636 |
| 57.0000 | 0.9091 | 0.4500 | 0.4091 | 23.3182 |
| 58.0000 | 0.9091 | 0.5000 | 0.4545 | 26.3636 |
| 59.0000 | 0.9091 | 0.5000 | 0.4545 | 26.8182 |
| 60.0000 | 0.9091 | 0.6875 | 0.6250 | 37.5000 |
| 61.0000 | 0.9091 | 0.5000 | 0.4545 | 27.7273 |
| 62.0000 | 0.9091 | 0.4615 | 0.4196 | 26.0140 |
| 63.0000 | 0.9091 | 0.5385 | 0.4895 | 30.8392 |
| 64.0000 | 0.9091 | 0.5556 | 0.5051 | 32.3232 |
| 65.0000 | 0.9091 | 0.7500 | 0.6818 | 44.3182 |
| 66.0000 | 0.9091 | 1.0000 | 0.9091 | 60.0000 |
| 67.0000 | 0.9091 | 0.5000 | 0.4545 | 30.4545 |
| 68.0000 | 0.9091 | 1.0000 | 0.9091 | 61.8182 |
| 69.0000 | 0.9091 | 0.0000 | 0.0000 | 0.0000 |
| 70.0000 | 0.9091 | 1.0000 | 0.9091 | 63.6364 |
| 71.0000 | 0.9091 | 0.0000 | 0.0000 | 0.0000 |
| 72.0000 | 0.9091 | 0.0000 | 0.0000 | 0.0000 |
| 73.0000 | 0.9091 | 0.0000 | 0.0000 | 0.0000 |
| 74.0000 | 0.9091 | 0.0000 | 0.0000 | 0.0000 |
| 75.0000 | 0.9091 | 0.0000 | 0.0000 | 0.0000 |
| 76.0000 | 0.9091 | 0.0000 | 0.0000 | 0.0000 |
| 77.0000 | 0.9091 | 0.0000 | 0.0000 | 0.0000 |
| 78.0000 | 0.8636 | 0.0000 | 0.0000 | 0.0000 |
| 79.0000 | 0.8182 | 0.0000 | 0.0000 | 0.0000 |
| 80.0000 | 0.7727 | 0.0000 | 0.0000 | 0.0000 |
| 81.0000 | 0.7273 | 0.0000 | 0.0000 | 0.0000 |
| 82.0000 | 0.6818 | 0.0000 | 0.0000 | 0.0000 |
| 83.0000 | 0.5909 | 0.0000 | 0.0000 | 0.0000 |
| 84.0000 | 0.4091 | 0.0000 | 0.0000 | 0.0000 |
| 85.0000 | 0.2727 | 0.0000 | 0.0000 | 0.0000 |
| 86.0000 | 0.2273 | 0.0000 | 0.0000 | 0.0000 |
| 87.0000 | 0.1364 | 0.0000 | 0.0000 | 0.0000 |
| 88.0000 | 0.0455 | 0.0000 | 0.0000 | 0.0000 |
| 89.0000 | 0.0455 | 0.0000 | 0.0000 | 0.0000 |
| 90.0000 | 0.0000 | 0.0000 | 0.0000 | 0.0000 |
| Σ |  |  | 66.4380 | 2342.5728 |

**Table 2** The life table of *Stratiolaelaps scimitus* feeding on *Tyrophagus putrescentiae* (25±1℃, 80%R.H., Dark)

| Lifetime x (d) | Survival rate l_x_ | Average production of female m_x_ | l_x_m_x_ | xl_x_m_x_ |
| --- | --- | --- | --- | --- |
| 1.0000 | 1.0000 | immature | immature | immature |
| 2.0000 | 1.0000 | immature | immature | immature |
| 3.0000 | 1.0000 | immature | immature | immature |
| 4.0000 | 0.9811 | immature | immature | immature |
| 5.0000 | 0.9811 | immature | immature | immature |
| 6.0000 | 0.9623 | immature | immature | immature |
| 7.0000 | 0.9623 | immature | immature | immature |
| 8.0000 | 0.9434 | immature | immature | immature |
| 9.0000 | 0.9434 | immature | immature | immature |
| 10.0000 | 0.9434 | immature | immature | immature |
| 11.0000 | 0.9245 | 0.0000 | 0.0000 | 0.0000 |
| 12.0000 | 0.9245 | 0.0000 | 0.0000 | 0.0000 |
| 13.0000 | 0.9245 | 0.0000 | 0.0000 | 0.0000 |
| 14.0000 | 0.9245 | 1.5000 | 1.3868 | 19.4145 |
| 15.0000 | 0.9245 | 1.3333 | 1.2327 | 18.4900 |
| 16.0000 | 0.9245 | 1.8947 | 1.7517 | 28.0269 |
| 17.0000 | 0.9245 | 1.1500 | 1.0632 | 18.0740 |
| 18.0000 | 0.9245 | 1.7500 | 1.6179 | 29.1218 |
| 19.0000 | 0.9245 | 1.3500 | 1.2481 | 23.7134 |
| 20.0000 | 0.9245 | 1.6500 | 1.5254 | 30.5085 |
| 21.0000 | 0.9245 | 1.9500 | 1.8028 | 37.8583 |
| 22.0000 | 0.9245 | 1.7000 | 1.5717 | 34.5763 |
| 23.0000 | 0.9245 | 1.8500 | 1.7103 | 39.3375 |
| 24.0000 | 0.9245 | 1.5000 | 1.3868 | 33.2820 |
| 25.0000 | 0.9245 | 1.7000 | 1.5717 | 39.2913 |
| 26.0000 | 0.9245 | 1.9000 | 1.7566 | 45.6703 |
| 27.0000 | 0.9245 | 1.6000 | 1.4792 | 39.9384 |
| 28.0000 | 0.9245 | 1.9000 | 1.7566 | 49.1834 |
| 29.0000 | 0.9245 | 1.3500 | 1.2481 | 36.1942 |
| 30.0000 | 0.9245 | 1.4500 | 1.3405 | 40.2158 |
| 31.0000 | 0.9245 | 1.7500 | 1.6179 | 50.1541 |
| 32.0000 | 0.9245 | 1.4500 | 1.3405 | 42.8968 |
| 33.0000 | 0.9245 | 1.3500 | 1.2481 | 41.1865 |
| 34.0000 | 0.9245 | 1.5500 | 1.4330 | 48.7212 |
| 35.0000 | 0.9245 | 1.1500 | 1.0632 | 37.2111 |
| 36.0000 | 0.9245 | 1.2500 | 1.1556 | 41.6025 |
| 37.0000 | 0.9245 | 1.0500 | 0.9707 | 35.9168 |
| 38.0000 | 0.9245 | 1.4000 | 1.2943 | 49.1834 |
| 39.0000 | 0.9245 | 0.9500 | 0.8783 | 34.2527 |
| 40.0000 | 0.9245 | 1.3500 | 1.2481 | 49.9230 |
| 41.0000 | 0.9245 | 1.5500 | 1.4330 | 58.7520 |
| 42.0000 | 0.9245 | 1.4500 | 1.3405 | 56.3021 |
| 43.0000 | 0.9245 | 1.0000 | 0.9245 | 39.7535 |
| 44.0000 | 0.9245 | 1.7500 | 1.6179 | 71.1865 |
| 45.0000 | 0.9245 | 0.8500 | 0.7858 | 35.3621 |
| 46.0000 | 0.9245 | 1.1000 | 1.0170 | 46.7797 |
| 47.0000 | 0.9245 | 1.2500 | 1.1556 | 54.3144 |
| 48.0000 | 0.9245 | 0.8000 | 0.7396 | 35.5008 |
| 49.0000 | 0.9245 | 1.0500 | 0.9707 | 47.5655 |
| 50.0000 | 0.9245 | 0.8500 | 0.7858 | 39.2913 |
| 51.0000 | 0.9245 | 0.9500 | 0.8783 | 44.7920 |
| 52.0000 | 0.9245 | 0.7500 | 0.6934 | 36.0555 |
| 53.0000 | 0.9245 | 0.7500 | 0.6934 | 36.7489 |
| 54.0000 | 0.9245 | 0.6000 | 0.5547 | 29.9538 |
| 55.0000 | 0.9245 | 0.8000 | 0.7396 | 40.6780 |
| 56.0000 | 0.9245 | 0.8500 | 0.7858 | 44.0062 |
| 57.0000 | 0.9245 | 0.4500 | 0.4160 | 23.7134 |
| 58.0000 | 0.9245 | 0.5263 | 0.4866 | 28.2216 |
| 59.0000 | 0.9245 | 0.8421 | 0.7785 | 45.9331 |
| 60.0000 | 0.9245 | 0.5556 | 0.5136 | 30.8167 |
| 61.0000 | 0.9245 | 0.6250 | 0.5778 | 35.2466 |
| 62.0000 | 0.9245 | 0.6154 | 0.5689 | 35.2732 |
| 63.0000 | 0.9245 | 0.7273 | 0.6724 | 42.3589 |
| 64.0000 | 0.9245 | 0.4444 | 0.4109 | 26.2969 |
| 65.0000 | 0.9245 | 0.6667 | 0.6163 | 40.0617 |
| 66.0000 | 0.9245 | 0.5000 | 0.4623 | 30.5085 |
| 67.0000 | 0.9245 | 0.6000 | 0.5547 | 37.1649 |
| 68.0000 | 0.9245 | 1.0000 | 0.9245 | 62.8660 |
| 69.0000 | 0.9245 | 0.0000 | 0.0000 | 0.0000 |
| 70.0000 | 0.9245 | 0.0000 | 0.0000 | 0.0000 |
| 71.0000 | 0.9245 | 0.0000 | 0.0000 | 0.0000 |
| 72.0000 | 0.9245 | 0.0000 | 0.0000 | 0.0000 |
| 73.0000 | 0.9245 | 0.0000 | 0.0000 | 0.0000 |
| 75.0000 | 0.9245 | 0.0000 | 0.0000 | 0.0000 |
| 76.0000 | 0.9245 | 0.0000 | 0.0000 | 0.0000 |
| 77.0000 | 0.9245 | 0.0000 | 0.0000 | 0.0000 |
| 78.0000 | 0.9245 | 0.0000 | 0.0000 | 0.0000 |
| 79.0000 | 0.9112 | 0.0000 | 0.0000 | 0.0000 |
| 80.0000 | 0.9112 | 0.0000 | 0.0000 | 0.0000 |
| 81.0000 | 0.9112 | 0.0000 | 0.0000 | 0.0000 |
| 82.0000 | 0.9000 | 0.0000 | 0.0000 | 0.0000 |
| 83.0000 | 0.8000 | 0.0000 | 0.0000 | 0.0000 |
| 84.0000 | 0.6500 | 0.0000 | 0.0000 | 0.0000 |
| 85.0000 | 0.5500 | 0.0000 | 0.0000 | 0.0000 |
| 86.0000 | 0.4500 | 0.0000 | 0.0000 | 0.0000 |
| 87.0000 | 0.3000 | 0.0000 | 0.0000 | 0.0000 |
| 88.0000 | 0.3000 | 0.0000 | 0.0000 | 0.0000 |
| 89.0000 | 0.2500 | 0.0000 | 0.0000 | 0.0000 |
| 90.0000 | 0.1500 | 0.0000 | 0.0000 | 0.0000 |
| 91.0000 | 0.0000 | 0.0000 | 0.0000 | 0.0000 |
| Σ | 0.0000 |  | 59.7974 | 2149.4481 |
